# Next generation highly resistant mirrors featuring all-silica layers

**DOI:** 10.1038/s41598-017-11275-0

**Published:** 2017-09-07

**Authors:** Tomas Tolenis, Lina Grinevičiūtė, Linas Smalakys, Mindaugas Ščiuka, Ramutis Drazdys, Lina Mažulė, Rytis Buzelis, Andrius Melninkaitis

**Affiliations:** 1 State Research Institute Center for Physical Sciences and Technology, Savanorių ave. 231, LT-02300 Vilnius, Lithuania; 2 Vilnius University, Laser Research Center, Saulėtekio ave. 10, LT-10223 Vilnius, Lithuania; 3 Lidaris UAB, Šv. Stepono st. 27C-24, LT-01312 Vilnius, Lithuania

## Abstract

A principal possibility to overcome fundamental (intrinsic) limit of pure optical materials laser light resistance is investigated by designing artificial materials with desired optical properties. We explore the suitability of high band-gap ultra-low refractive index material (*n* less than 1.38 at 550 nm) in the context of highly reflective coatings with enhanced optical resistance. The new generation all-silica (porous/nonporous) SiO_2_ thin film mirror with 99% reflectivity was prepared by glancing angle deposition (GLAD). Its damage performance was directly compared with state of the art hafnia/silica coating produced by Ion-Beam-Sputtering. Laser-Induced Damage Thresholds (LIDT) of both coatings were measured in nanosecond regime at 355 nm wavelength. Novel approach indicates the potential for coating to withstand laser fluence of at least 65 J/cm^2^ without reaching intrinsic threshold value. Reported concept can be expanded to virtually any design thus opening a new way of next generation thin film production well suited for high power laser applications.

## Introduction

Laser-induced damage (LID) phenomena is a principal limitation preventing achieval of higher optical power in almost every modern laser facility. Soon after the discovery of lasers LID was observed in transparent dielectric material^1^. The problem of optical damage rippled throughout the years inspiring novel concepts of optics fabrication as well as ways to characterize so-called Laser-Induced Damage Threshold (LIDT) parameter^2^. Nowadays LID still remains a primary reason why modern high power lasers are exceeding sizes of the table top and are rather comparable with football stadium: further increase of optical average- and peak- power is now only possible by the expansion of laser beam sizes and the combination of multiple laser beams into one focal point. Accordingly, expensive large size (meter scale) critical laser elements are needed in such laser facilities as National Ignition Facility^3^, LMJ^4^ or ELI^5^. Thus, any improvement in laser components ability to withstand more intense light could lead to either higher optical power or to smaller cost effective high power laser solutions that could deliver high power to real world applications.

LIDT has been studied extensively over many decades^6, 7^. Quite early it was understood that most of the optical components are always damaged at the fluence, much less than the intrinsic - bulk LIDT of the optical material. Various production defects have been identified as damage precursors that trigger the extrinsic LID at low fluence. Such defects can originate in any production step: starting from material synthesis and surface preparation^8^ or later introduced by deposition technology^9^. Defects cause either strong electric field intensity^10^ enhancement and/or additional absorptivity, which results in the enhanced optical energy deposition in the vicinity of defects and initiates LID. In recent years significant progress was achieved in surface defect minimization: special techniques of glass polishing^11^, post fabrication etching techniques^12^ as well as advanced thin film deposition^13, 14^ methods were developed. Aside from the defect minimization, efforts to improve intrinsic LIDT in optical coatings are also explored. First of all, an exhaustive search of natural materials with highest intrinsic damage resistance was done. Two types of correlations were found in the case of dielectric interference coatings and namely, LIDT is directly proportional to the bandgap of the constituting layers^15^ and inversely proportional to its refractive index^16^. Material mixing concept was used in IBS deposition process^17, 18^ in order to achieve better LIDT resistance by tuning material composition towards higher band-gap values. Additionall tailoring of electric field^19^ distribution inside the multilayer coating stack can be also applied by changing the thicknesses of substituting layers. It is possible to maintain appropriate spectral performance by shifting maximum peaks of the electric field towards more laser resistant layers^20, 21^ or by inserting layers with intermediate refractive index^22^. After such optimization, shifted peaks are typically located in more resistant low refractive index (high bandgap) layers, e.g. SiO_2_ thus increasing overall LIDT of the component. Once defect minimization, electric field optimization, and natural material choice limits will be exhausted, fundamental - intrinsic laser damage resistance limit will be reached. Next and only one yet known possibility to push materials resistance limit further is designing and application of artificial materials with ultra-low refractive index (*n* less than 1.38 at 550 nm). A similar approach was recently demonstrated for single layer anti-reflection coatings by etching the substrates and forming porous thin film^23^ with LID threshold close to the same of pure glass^24^. The formation of all-silica multilayer anti-reflection coating has been recently reported^25^. In both cases, the effort is taken to reduce refractive index and laser radiation intensity at the weakest link of the multilayer coating.

Although the concept of reflecting coatings consisting of porous-nonporous layers was already demonstrated^26^, their potential has not yet been explored in the context of laser damage.

The objective of this research was to determine whether such coating design in combination of the low refractive index - high bandgap material has a potential that could lead to conceptually novel route how laser components for high power facilities are engineered. For our case study, we chose SiO_2_ as a popular high bandgap material in order to produce high reflection Bragg mirror for UV region. The porosity modulation was achieved via GLancing Angle Deposition method (GLAD) by varying angle of incidence of incoming vapor particles with respect to the substrate surface normal. Optical and structural properties of the produced experimental mirror are characterized and directly compared with a similar multilayer coating, produced by state of the art Ion Beam Sputtering (IBS) technology and consisting of classical HfO_2_ and SiO_2_ layer design.

## Production of the samples

Six types of experimental samples were deposited in total. In order to characterize refractive indexes of evaporated materials, four single layers were fabricated on fused silica (FS) substrates using both IBS and e-beam evaporation technologies. Afterward, two high reflectivity mirrors were formed as an investigation subjects of this work.

Great care was taken for preparation of FS substrates before the start of the evaporation process. Firstly, substrates were mechanically cleaned with cotton swabs and alcohol. Then ultrasonic cleaning in an aqueous surfactant with alkaline cleaning solution was used. Finally, the substrates were rinsed twice in distilled warm water and dried with IR radiation. Identical substrates were used for all deposition processes.

Dense HfO_2_ and SiO_2_ single layer films of 500 nm physical thickness were sputtered using Cutting Edge Coatings (Germany) IBS coating plant. Oxide materials were synthesized in a vacuum chamber (10^−4^ mbar) by sputtering Hf and Si metal targets and injecting O_2_ flow at 8 sccm (standard cubic centimeters per minute) and 50 sccm rates for Hf and Si materials, respectively. Films thickness were controlled by the broadband optical monitoring, which constantly measures the transmission of the deposited layer, fits the theoretical model and cuts the deposition process when necessary thickness is coated. The sputtering rates during the process were maintained at 0.6 Å/s and 1.2 Å/s for HfO_2_ and SiO_2_ materials, respectively.

**Figure 1:**
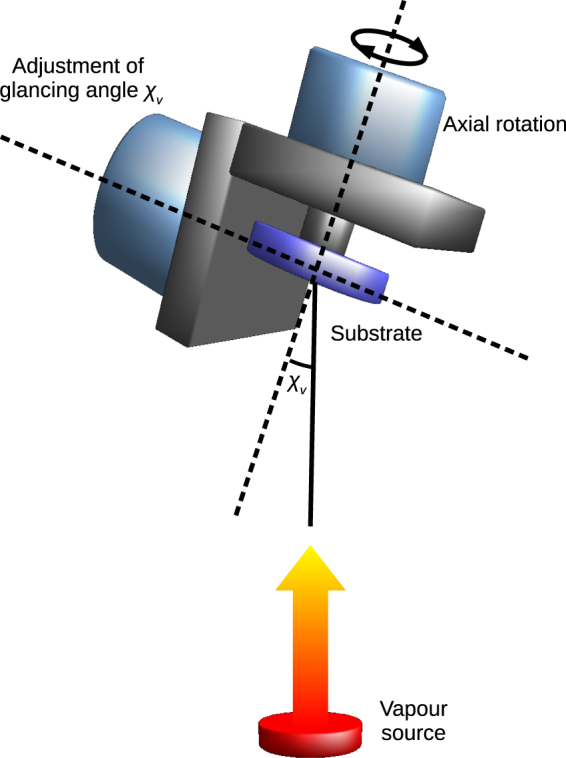
Principal scheme of glancing angle deposition: *χ*_*v*_ between the vapour source and rotating substrate normal is set at acute angle.

All-silica thin films of modulated porosity were deposited using Sidrabe (Latvia) e-beam evaporation plant, equipped with electron beam source and two stepper motors system (see Fig. 1). The substrates were placed in substrate holder rotating around its axis. The second rotation axis was used to control the angle between vapor source and the substrate normal. Two single-layers of 500 nm physical thickness were deposited by evaporating SiO_2_ material and changing only the angle of deposition at 0° and 70° accordingly. The deposition rate maintained at 3 Å/s for both angles while the thickness of each film was controlled by quartz crystal monitor, placed near the substrates. For every deposition angle, the substrate holder was rotated around its axis at the speed of 0.5 revolutions per second to maintain the constant thickness distribution over the glasses as well as suppress birefringence effects well known for sculptured thin films^27^.

**Figure 2:**
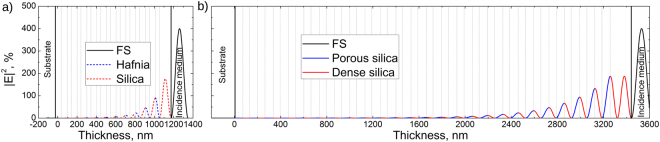
E-field distributions in multilayer high reflection samples, modelled by (**a**) dense HfO_2_ and SiO_2_ materials, and (**b**) porous and dense SiO_2_ materials. Blue and red dashed lines represent dense HfO_2_ and SiO_2_ materials, respectively, produced by IBS process. Blue and red solid lines represent porous and dense SiO_2_, respectively, produced by GLAD process.

As the second step, two multilayer coatings were designed to reflect 99.5% of the incident light at 355 nm wavelength and 0° angle of incidence. Quarter wave optical thickness layers were used as building blocks to form multilayers. The design of 22 layers was used to form high reflection mirror of dense HfO_2_ (H–high index) and SiO_2_ (L–low index) materials. The thicknesses were 42 nm and 59 nm for high and low refractive index thin films, respectively. The double quarter optical thickness of low refractive index material (2L), namely silica, was used as the last layer in the design for better resistance to laser radiation^28^. The total designed physical thickness of multilayer was 1172 nm. In the case of dense (H) and porous (L) silica reflector, featuring relatively low refractive index contrast, 50 layers were required in total to achieve a theoretical reflectivity of 99%. The thicknesses of high and low refractive index thin films were 61 nm and 72 nm, respectively. The coating would suffer from mechanically weak porous film on top when using the same optical design of last 2L layer for all-silica mirror, thus, triple quarter (3H) optical thickness of dense silica was used as the last layer to maintain reflectivity and better mechanical resistance of the mirror. The total designed physical thickness of all-silica multilayer was 3442 nm. The modeled distributions of the electric field in both mirror samples are presented in Fig. 2. Distributions of electric field intensity are standard for the quarter-based mirror design. However, the penetration depth is very different. The decrease of intensity by 50% within the coating is reached after light passes approximately through 2 and 5 layers in IBS355 and GLAD355 samples, respectively.

First experimental mirror, named IBS355, was fabricated using ion-beam sputtering technology. HfO_2_ and SiO_2_ layers were deposited using aforementioned process conditions as for single layers. For complete oxidation after the sputtering process, multilayer coating was annealed in the atmosphere for 2 hours at 300 °C. Sample IBS355 was considered as a standard high reflection mirror, resistant to laser radiation.

Second experimental mirror, named GLAD355, was fabricated by the GLAD method. Only SiO_2_ material was evaporated using e-beam. Aforementioned formation process for porous and dense SiO_2_ material was used to form an experimental sample of 50 layers. No heating of the chamber was used during or after the process.

## Results

The main results of investigation are presented in the following table:

**Table:** 

**Main results of investigation**				
**Sample**	Reflectivity	Light scattering (TIS)	Surface roughness	Intrinsic LIDT
**IBS355**	99.5%	0.072%	0.5 nm	32 J/cm^2^
**GLAD355**	98.6%	0.72%	2.58 nm	>65 J/cm^2^

The complete analysis of defined characteristics are discussed in the next sections.

### Optical characterization

**Figure 3:**
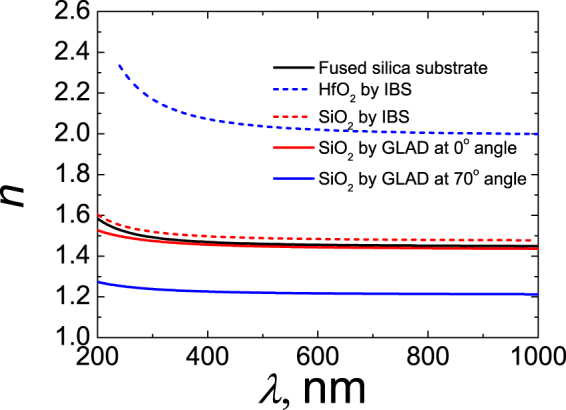
Refractive index dispersions of FS substrate and thin films produced by IBS and GLAD methods.

Dispersions of single layer thin film refraction indexes are presented in Fig. 3. HfO_2_ deposited by IBS is considered as the reference high refractive index layer material in standard quarter-stack based high reflectivity mirror. It is known for its high resistivity to laser irradiation^29^. The refractive index of hafnia at the wavelength of 355 nm is 2.1. SiO_2_ was an obvious choice for low refractive index material since its refractive index is 1.51 at the same wavelength for IBS technology. In the case of GLAD, different refractive indexes of silica single-layers were evaluated for two evaporation angles. The dense SiO_2_ thin film, with a refractive index of 1.47, has been evaporated at *χ*_*v*_ = 0° angle. The refractive index is lower than in silica thin film, produced by IBS technology. Denser coatings are fabricated using sputtering process compared to evaporation technique, because of more energetic nature of vapor stream^30^. The porous SiO_2_ thin film, with a refractive index of 1.23, has been evaporated at *χ*_*v*_ = 70° angle. The porosity was induced by self-shadowing effect^31^. Constant rotation around an axis of the substrate enabled to form circular cross-section columns with no distinct increase in radius. No birefringence has been detected in the porous thin film by measuring optical response at normal light incidence angle. Thin films with refractive indexes close air index can be fabricated when deposition angle approaches 90°^32^. The GLAD355 samples were produced by evaporating only SiO_2_ material at 0° and 70°. The difference between refractive indexes of evaporated dense and porous silica single layers is 0.24. Such value is lower than the difference between sputtered HfO_2_ and SiO_2_ indexes (0.59) but sufficient for formation of Bragg mirror.

**Figure 4:**
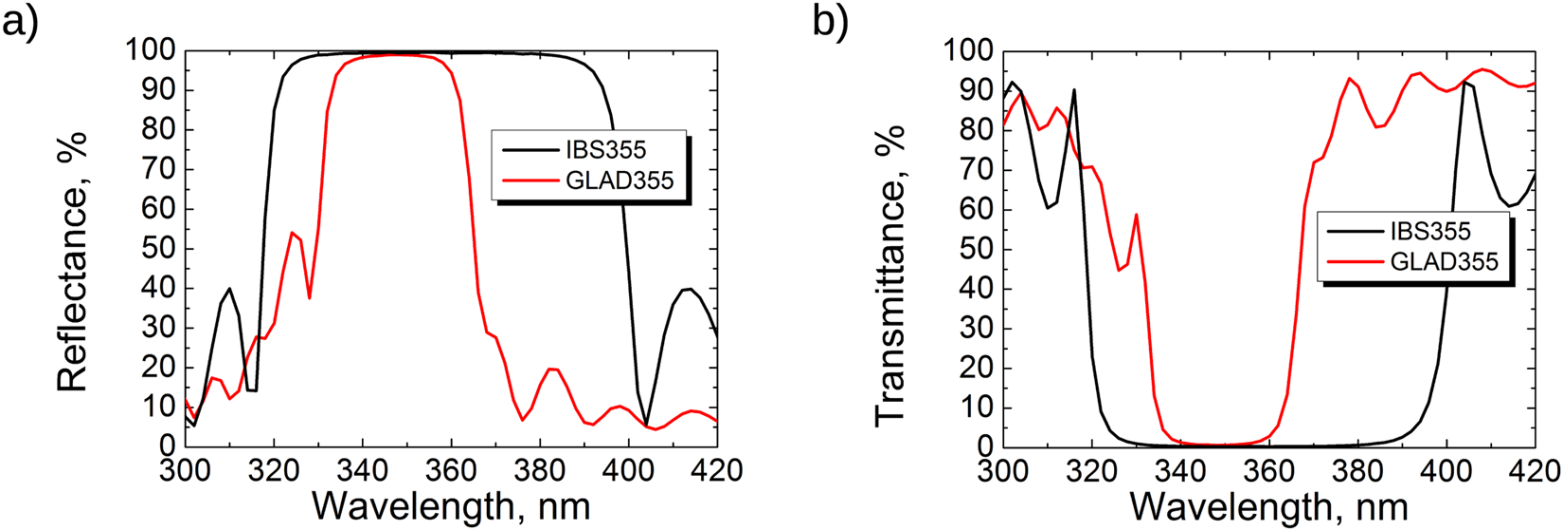
(**a**) Reflection and (**b**) transmission measurements of high reflection mirror samples IBS355 and GLAD355.

For the incidence angle of 8°, experimental light reflection spectra of multi-layer mirrors IBS355 and GLAD355 are presented in Fig. 4. Observing reflection spectra, it is evident that reflection bandwidth of IBS355 sample is wider than GLAD355 sample. Such behavior can be explained by the difference of high and low refractive indexes in GLAD355 sample: contrast is lower than in IBS355, therefore the reflection band is narrower. The width of sputtered mirror at 90% reflectivity level is 74 nm when evaporated GLAD mirror is more than twice narrower and covers 28 nm of reflection. Also, the reflection peak for GLAD355 sample reaches *R* ≈ 98.6%, when for IBS355 sample it is *R* ≈ 99.5%. The transmission values are 0.9% and 0.3% for GLAD355 and IBS355 samples, respectively. Therefore, optical losses of approximately 0.5% for the all-silica mirror was registered. Since silica has a low absorption in both UV and VIS spectral regions, optical scattering was considered as a candidate of optical losses.

### Scattering measurements

**Figure 5:**
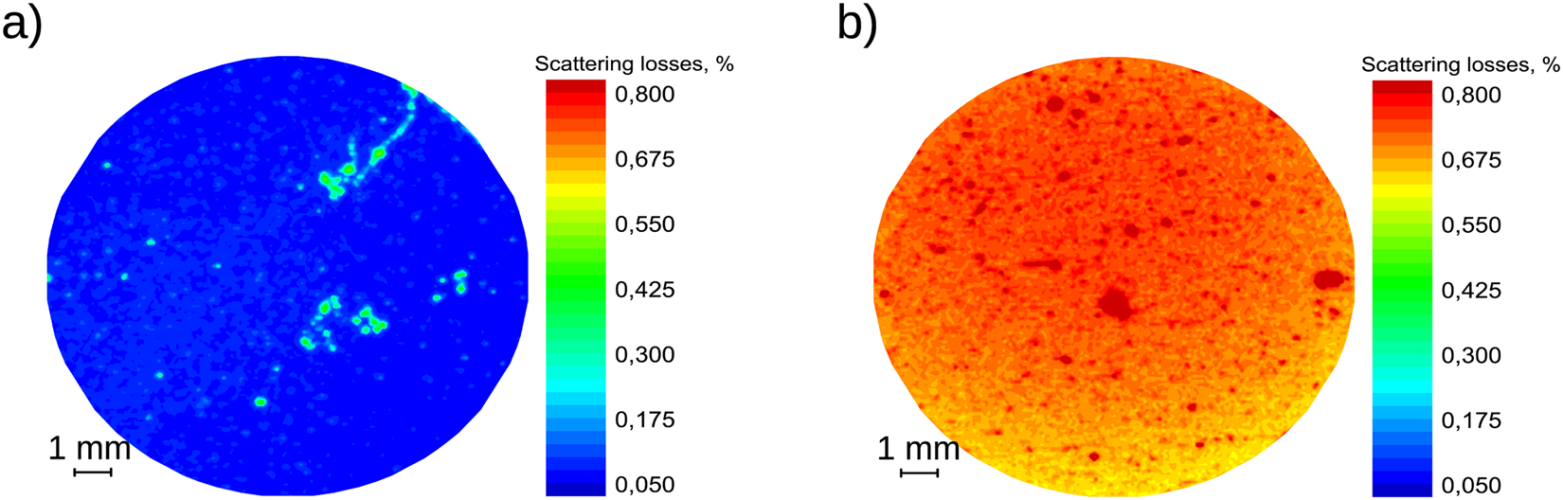
The maps of total integrated scattering losses for (**a**) IBS355 and (**b**) GLAD355 samples.

Light scattering maps are presented in Fig. 5. The median estimated values of TIS (Total Integrated Scattering) were 0.72% and 0.072% for GLAD355 and IBS355 samples, respectively. GLAD mirror exhibited ten times larger scattering values than the mirror, produced by sputtering technology. Localized scattering spikes are visible in both maps. Surface roughness and/or structural volumetric inhomogeneities are common factors in Rayleigh scattering. Sources of scattering in IBS355 coating appear due to substrate scratches left either from polishing or manual cleaning while dotted spikes most likely are generated by nodular defects. It is worth mentioning, that most of the laser damage testing was performed in defect-free low scattering area. The significantly higher quantity of defects also of larger size can be seen as scattering spikes for the GLAD355 sample. Surface imperfections, bulk scattering in porous layers as well as nodular defects are assumed sources of scattering losses. Additional microscopic analyses (surface roughness and apparent defect density measurements) were executed to verify those assumptions as significant scattering losses in high reflection all-silica mirror is a considerable disadvantage which must be addressed in the future research.

### AFM measurements

**Figure 6:**
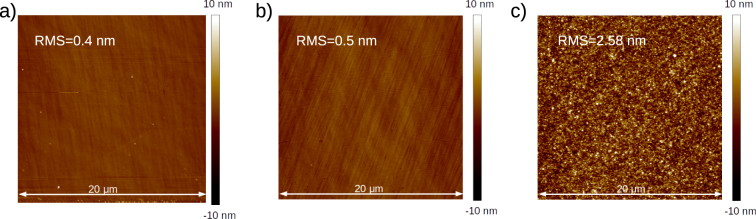
AFM images of (**a**) bare FS substrate, (**b**) IBS355 sample and (**c**) GLAD355 sample.

AFM measurements indicate, that the RMS (root mean square) value of substrate surface roughness was 0.4 nm (see Fig. 6). The roughness of IBS355 and GLAD355 coatings were 0.5 nm and 2.5 nm, respectively. Ion beam sputtering technology is known for its ability to coat the substrates conformally due to highly energetic vapor particles^18^. And indeed smooth surface was obtained in the case of the sputtering process. The surface roughness of evaporated SiO_2_ thin film on smooth glass can reach 1.4 nm^33^. Relatively high surface roughness was measured for GLAD355, thus confirming the fact that material deposition at a glancing angle can increase the surface roughness even tenfold^34^. Significant optical losses for the GLAD355 sample are consistent with surface roughness measurements, which indicated five times larger RMS value for the same sample if compared to the values of IBS355 sample or the substrate. Surface roughness can be used as a good predictor of light scattering when using^35^ formalism. Estimated light scattering values of 0.77% and 0.03% were obtained for GLAD355 and IBS355 mirrors, respectively, which is in agreement with TIS measurements. Therefore, large surface roughness can be considered as the main source of optical losses for multilayer mirrors, produced by the GLAD method. The origins of such surface irregularities can be explained by columnar nature of inner layer morphology of the coating. TIS maps also indicate the presence of possible large structural defects which are further investigated in detail by SEM and Optical microscopies.

### SEM measurements

**Figure 7:**
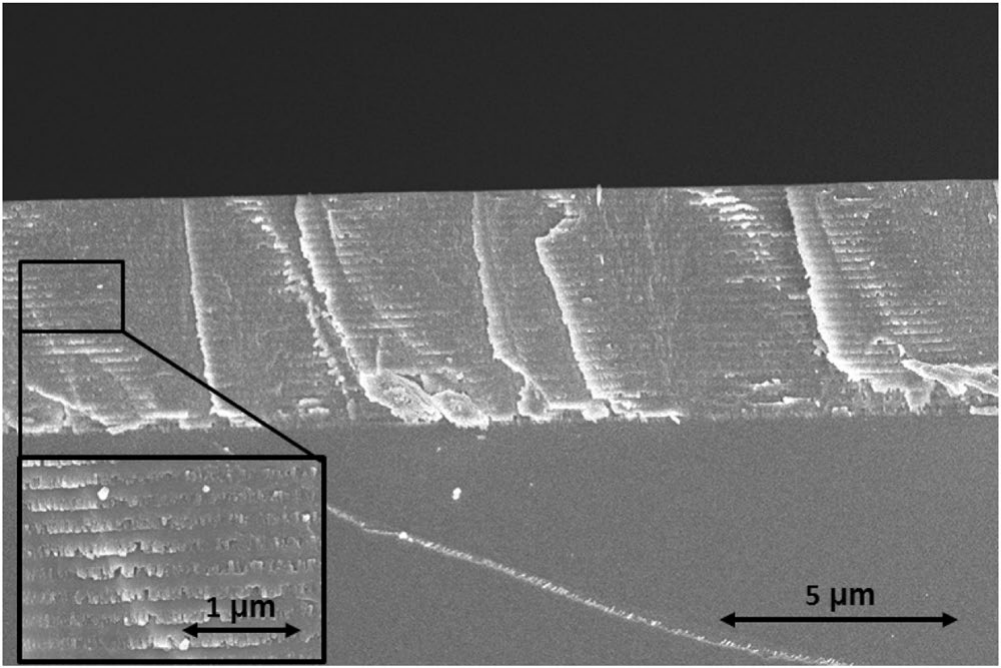
Cross sectional SEM image of GLAD355 sample.

SEM analysis, conducted to study GLAD355 mirror’s cross-section and inner structure, is shown in Fig. 7. The distinct layers are apparent inside multilayer coating structure indicating changes from porous to dense states. The porous structure consists of nano-columnar morphology known for sculptured thin films when vapor stream approaches substrate at a glancing angle while it is rotated around an axis normal to the surface^36^. As it is seen from Fig. 7, dense layers conformally coat columnar structure, thus, possibly repeat their surface irregularities and cause light scattering.

### Laser damage resistance and optical microscopy

**Figure 8:**
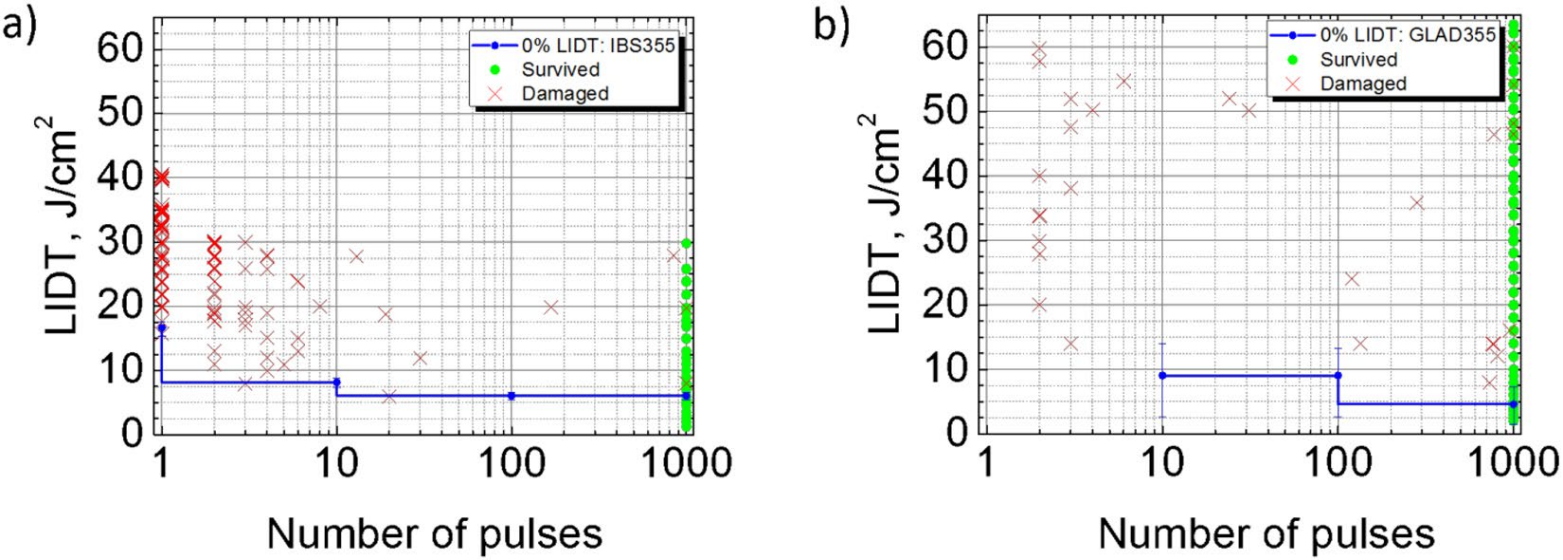
The results of laser damage resistance testing for (**a**) IBS355 and (**b**) GLAD355 experimental samples.

The results of laser damage resistance testing are plotted in Fig. 8 for both mirrors. X-shaped points represent the number of laser pulses required to trigger catastrophic failure events on unexposed sites with given laser fluence. Circles indicate censored “no damage” events after maximal exposure with 1000 laser pulses. In the case of single shot irradiation (1-on-1 testing mode) no damage event was detected with maximal available laser fluence of 65 J/cm^2^ for GLAD355 sample, while 16.7 J/cm^2^ was measured for the conventional IBS355 mirror. In the case of multi-shot irradiation (1000-on-1 testing mode) both samples showed quite low extrapolated LIDTs: 6.0 and 4.6 J/cm^2^ for IBS355 and GLAD355 mirrors respectively. Both 1-on-1 and S-on-1 LIDT values of reference hafnia-silica IBS sample correlate well with the data from scientific literature: (5–12 J/cm^2^)^37, 38^ and (5–10 J/cm^2^)^37^, respectively, obtained under similar irradiation conditions.

**Figure 9:**
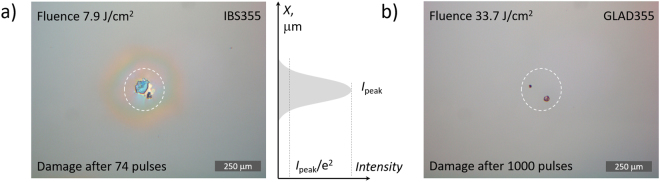
(**a**) DIC microscopy (10x) image representing typical damage morphologies for IBS355 (**a**) GLAD355 (**b**) coatings. A 200 *μ*m dashed circle represents irradiated spot size of Gaussian laser beam.

As can be seen from microscopic damage morphology pictures in Fig. 9 initiation of damage always starts at localized extrinsic defect site. In most of the cases, damage occurred when laser illuminated the nodular defect with a fluence exceeding the local threshold. In the case of IBS355 mirrors 6 J/cm^2^ was sufficient to initiate first localized damage pinpoints that most likely can be attributed to embedded metallic hafnium nano-clusters^39^.

Densities of apparent nodular defects were estimated prior to LIDT testing: 15 defect/mm^2^ and 55 defect/mm^2^ for the IBS355 and GLAD355 samples, respectively. It is highly probable that the same defects also significantly contribute to optical scattering losses.

**Figure 10:**
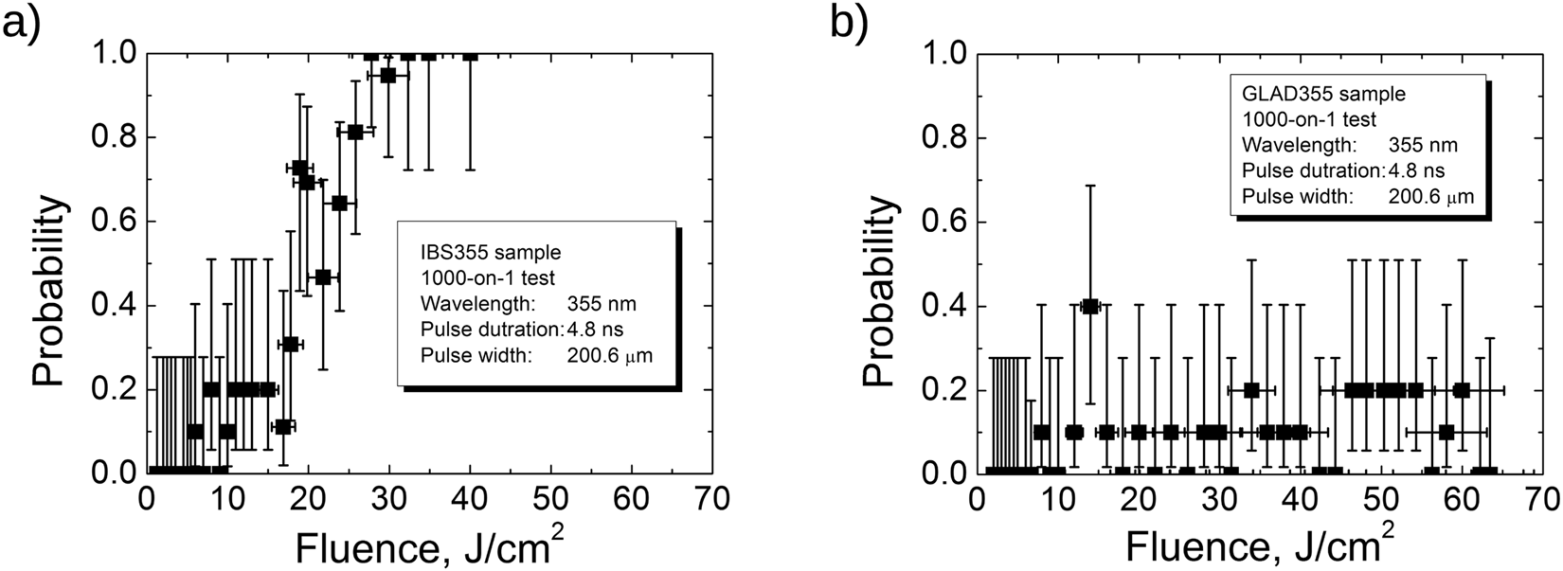
Probabilities of laser damage versus peak fluence level in 1000-on-1 regime for (**a**) IBS355 and (**b**) GLAD355 experimental samples.

Damage probability results corresponding to 1000-on-1th laser pulse class are extracted into separate graphs (Fig. 10). A sudden increase in probability is observed for the IBS355 sample between 6 J/cm^2^ and 32 J/cm^2^. A 100% damage probability is reached at a fluence level of 32 J/cm^2^ and is very likely a good indicator of a fundamental limitation of hafnia silica matrix representing LIDT without defects, nevertheless it is worth mentioning that an intrinsic LIDT could be also underestimated because of dependence on spot size and defect density. The intrinsic resistance of conventional multilayer coating is limited by the layers with lowest intrinsic LIDT^22^. In case of IBS355 sample every H layer is formed out of low band gap material HfO_2_ (5.5 eV^40^) while dense SiO_2_ with higher band-gap was used as H material for GLAD355 coating. The latter has considerably higher intrinsic laser resistance than hafnia^41^. And indeed, in the case of all-silica mirror laser damage probability looks very different regardless of laser fluence as shown in Fig. 10b). As indicated by analysis of microscopy images, density of apparent defects is much higher in the GLAD355 sample thus the damage probability curve is also expected to be dominated by defect induced failure mechanism. In damage morphology craters localized precursors can be seen (they are first initiated at 8 J/cm^2^). Surprisingly, highest damage probabilities (except one outlier of 40% observed at 14 J/cm^2^) do not exceed 20% even for fluence as high as 65 J/cm^2^, (the highest available fluence of the measurement system) thus indicating outstanding potential intrinsic LIDT value.

## Discussion

A classical paradigm of how thin films for high power lasers are designed and produced is reconsidered by collecting all the previous knowledge from recent experimental findings. Accordingly, a principal possibility to overcome intrinsic laser damage resistance, limited by dense high refractive index metal-oxide materials, was investigated for optical interference coatings. A novel approach was proposed: instead of using classical dense HL layers, alternating porous ultra-low refractive index- and dense low index material layers were deposited out of the high band-gap single material source. A new generation all-silica mirror was successfully realized by using so-called glancing angle deposition method in combination with electron beam evaporation. Although the designed reflectivity (99.5%) of the all-silica mirror was not yet achieved due to significant scattering losses (0.728%) and high surface roughness (2.5 nm, RMS), the novel approach demonstrates high potential for intrinsic laser damage resistance. The damage performance of all-silica mirror was directly compared with state of the art hafnia-silica coating produced by Ion-Beam-Sputtering. The damage resistance of both coatings was tested (1000-on-1 protocol) in nanosecond regime at 355 nm wavelength and showed similar performance limited by extrinsic defect precursors. Nevertheless, all silica coating exhibited only 20% damage probability at 65 J/cm^2^ thus confirming the potential superiority of proposed approach.

The observed differences between the intrinsic and the extrinsic LIDT’s in all-silica coatings can be better understood by exploring electric-field enhancement due to so called nodular defects of deposition process. Light can be intensified by factor as high as 24X and tends to increase with inclusion diameter. Irradiation also has a significant effect on light intensification within the defect and the multilayer^10^. Removal of such nodules by using advanced deposition methods^13, 14^ would help to mitigate losses in the all-silica-mirrors as the observed intrinsic damage resistance was demonstrated to be very high. High intrinsic LIDTs thus also confirm that proposed principle of tailored porosity in combination high bandgap deposition materials could lead to an entirely new class of highly resistant coatings for very demanding high power laser applications.

Although further research efforts are needed to minimize scattering losses and nodular defects, we believe that reported concept can be reproduced with other known designs of classical thin film coatings thus opening a new way of next generation optics well suited for high-power laser applications.

## Methods of characterization

Spectrophotometric transmission measurements were performed with spectrophotometer RTPhoton (Belarus). Transmission spectra of single-layers were analyzed in the low absorptance spectral region with commercial software “Optilayer”. Refractive indexes and thickness were modeled using Sellmeier equation and simulating Fresnel reflection and transmission spectra of substrate-layer optical system. Extracted refractive indexes were further used in designing high reflection mirrors and determining the thickness of constituent layers. “Optilayer” software was also used to evaluate electric field intensity distribution within high reflectivity multilayer coatings.

Mapping of Total Integrated Scattering (TIS) losses was performed at the wavelength of 355 nm by virtually dividing 1-inch aperture into the grid of 16031 hexagon matrix cells. Beam diameter (1/e^2^) in target plane was used to investigate every cell of the grid and set to 174 *μ*m while spatial resolution of the measurement was limited by the cell size parameter, which is the distance (d = 100 *μ*m) between centroids of neighboring cells. TIS measurement system is described in more detail by *Mažulė et al*.^42^.

The additional silicon substrate was placed in evaporation chamber for analysis of the films nanostructure by scanning electron microscope (SEM). After the coating process, samples were fractured and deposited with 20 nm of Cr layer by magnetron sputtering to avoid charging effect. The morphology of the samples was characterized using SEM workstation Helios Nanolab 650 (USA). The imaging of nanostructure was carried out under an accelerating voltage of 3 kV.

The surface roughness of the multilayer coatings was measured using atomic force microscope Dimension Edge of Bruker. Measurements were performed in tapping mode and in ambient conditions. Root mean square values of the surface roughness were determined by analyzing two 20 × 20 *μ*m^2^ surface areas with a scan resolution of 512 pixels × 512 pixels of both mirror coatings and the uncoated substrate.

The automated in-house built LIDT test bench, based on a single longitudinal mode injection seeded Nd:YAG laser system from EKSPLA (Lithuania), was used for laser damage resistance testing. Laser delivers linearly polarized pulses with full-width half-maximum (FWHM) of 8 ns at 1064 nm wavelength and is equipped with two nonlinear crystals, which generate pulses of 4.8 ns (FWHM) at 355 nm wavelength. Fluence is adjusted with a motorized attenuator, consisting of a half-wave plate and a polarizer. Laser pulse energy is monitored by a calibrated photodiode. Spatial beam profile is characterized before the measurement by a charge coupled device (CCD camera). The lateral pixel resolution of the CCD camera is 3.75 *μ*m. A mechanical shutter is employed in order to pick out laser burst from a pulse train with 100 Hz repetition frequency.

The laser-induced damage probability testing is performed at 0 degrees angle of incidence following standard 1000-on-1 ISO 21254-2 test procedure^2^. The surface of each sample is virtually divided into a matrix of sites to be tested with no overlap. Maximum of 1000 laser pulses are applied to each site at fluences of interest. A constant number of sites are irradiated at certain onset laser fluence. After the exposition, every site is inspected by differential interference contrast (Nomarski) microscopy and recorded as damaged or non-damaged. Then, damage probability is calculated as a ratio between damaged and all exposed sites at particular fluence. Next, onset fluence is increased and the same number of fresh sites is irradiated again. Following this algorithm, the whole optics surface is exposed. More than 30 different fluence levels were used for testing of at least 10 fresh sites for each fluence level, resulting in about 430 total test sites for each sample. Uncertainty of measured peak fluence (±6%) is limited by variation of lasers effective beam diameter and pulse to pulse energy stability. Probability error bars are estimated by using Bayesian approach and considering binomial nature of laser damage testing experiments (either damaged of non-damaged) at given peak fluence^43^. Both statistical and fluence error bars correspond to a confidence level of 95%. The damage threshold is estimated by maximum likelihood fitting algorithm of damage probability statistics versus applied fluence. LIDT evaluation procedure is described in reference^44^.

Finally, laser damage inspection, morphologies, and defects on the coating surfaces were observed with optical microscope Olympus BX41. Images were captured with PixeLink camera increasing the view by 20 times with MPlanFL N objective. Average sefect density was analyzed by registering 10 images in dark field regime, inverting them and analyzing with ImageJ software.
